# Parents’ Experiences and Clinicians’ Perceptions of Managing Cancer Pain in Young Children at Home

**DOI:** 10.3390/curroncol32100538

**Published:** 2025-09-26

**Authors:** Lindsay A. Jibb, Elham Hashemi, Surabhi Sivaratnam, Aimee K. Hildenbrand, Paul C. Nathan, Julie Chartrand, Nicole M. Alberts, Tatenda Masama, Hannah G. Pease, Lessley B. Torres, Haydee G. Cortes, Mallory Zworth, Susan Kuczynski, Michelle A. Fortier

**Affiliations:** 1 Lawrence Bloomberg Faculty of Nursing, University of Toronto, Toronto, ON M5T 1P8, Canada; 2 Child Health Evaluative Sciences, Hospital for Sick Children, Toronto, ON M5G 1X8, Canada; 3 Department of Pediatrics, Hospital for Sick Children, Toronto, ON M5G 1X8, Canada; 4 Center for Healthcare Delivery Science, Nemours Children’s Health, Delaware, DE 19803, USA; 5 Institute of Health Policy, Management, and Evaluation, University of Toronto, Toronto, ON M5T 3M6, Canada; 6 Faculty of Health Sciences, University of Ottawa, Ottawa, ON K1S 5S9, Canada; 7 Children’s Hospital of Eastern Ontario Research Institute, Ottawa, ON K1H 8L1, Canada; 8 Department of Psychology, Concordia University, Montreal, QC H3G 1M8, Canada; 9 Sue and Bill Gross School of Nursing, University of California Irvine, Irvine, CA 92697, USA; 10 Department of Pediatric Psychology, Children’s Hospital of Orange County, Orange, CA 92868, USA; 11 UCI Center on Stress and Health, School of Medicine, University of California Irvine, Orange, CA 92868, USA; 12 Ontario Parents Advocating for Children with Cancer, Toronto, ON M4G 1R8, Canada

**Keywords:** pain, children, parents, home-based care, qualitative

## Abstract

Background: Pain is a prevalent and distressing symptom for children with cancer, negatively affecting quality of life and family functioning. While most research focuses on hospital-based care, many pain episodes occur at home, where parents act as primary caregivers with limited access to evidence-based symptom management. Young children are particularly vulnerable due to limited self-reporting capacity and reliance on parental assessment. We aimed to explore parent experiences and pediatric oncology clinician perceptions of young children’s cancer pain at home, its impact on families, and recommended supports. Methods: Using an interpretive descriptive qualitative design, we conducted semi-structured interviews with parents of children aged 2–11 years undergoing outpatient cancer treatment and clinicians at two hospitals in Canada and the United States. Data were analyzed using thematic analysis. Results: In total, 21 parents and 21 clinicians participated. Three themes were developed: (1) the multifaceted experience of young children’s cancer pain at home, (2) the ripple effects of a young child’s cancer pain on the family unit, and (3) assessing and treating children’s cancer pain at home. Conclusion: Managing cancer pain at home places substantial emotional and practical demands on the families of young children. Our findings highlight that structured supports providing parents and clinicians with education, effective communication pathways, and collaboration opportunities may optimize home-based pain care, reduce caregiving burden, and improve outcomes for children and their families.

## Simple Summary

Managing cancer pain in young children at home places heavy emotional and practical demands on parents. Through interviews with parents and pediatric oncology clinicians, we found that children’s pain at home is frequent, disruptive to daily life, and distressing for families. Parents reported uncertainty about how to assess and treat pain, while clinicians highlighted workload constraints that limited the education and support they could provide. Despite these challenges, both groups emphasized the critical role of parents in recognizing and managing pain and the need for clearer communication pathways, structured education, and peer support. These findings suggest that strengthening hospital–home connections, including through digital and telemedicine-based tools, may improve pain management and reduce caregiver burden.

## 1. Introduction

Children with cancer report pain as one of the most common and distressing symptoms of their disease and treatment [1]. An extensive body of research shows that cancer pain negatively impacts child quality of life, impairs family functioning, and is a major reason for cancer-related emergency health service use in adult patients [2,3,4]. When children are outside of clinical environments, the responsibility of managing cancer pain and its negative impact largely lies with a child’s family, who sometimes provide this care with limited access to evidence-based treatments [5,6]. Although most childhood cancer pain research has been conducted in hospital settings, evidence indicates that significant percentages of children experience mild (~60%) or moderate to severe pain (~26%) at home [7].

Within this context, toddlers and school-aged children receiving outpatient cancer care are particularly vulnerable because of their developmental stage-related limited ability to self-report pain and a reliance on parents to know when and how to effectively deploy pain management interventions or seek medical treatment [8,9]. Empowering parents to assess and treat cancer pain in young children—through mechanisms that connect the hospital and home, including digital or telemedicine-based tools—may improve pain and family wellness [6,10,11]. While these issues suggest a need for system-level changes, a clearer understanding of how parents navigate pain at home and which services and supports are essential for effective caregiving is first needed. Accordingly, we aimed to describe the experiences of parents of young children with cancer as well as the perceptions of pediatric oncology clinicians as they pertained to children’s cancer pain experiences outside of the hospital, its impact on the family unit, current home-based pain management practices, and needs.

## 2. Methods

Our reporting is in accordance with the Consolidated Criteria for Reporting Qualitative Research (COREQ) [12] (Appendix A) and Guidance for Reporting Involvement of Patients and the Public—Short Form (GRIPP2-SF) [13]. 

### 2.1. Study Design, Setting, and Participants

We used an interpretive, descriptive, qualitative interview-based design [14]. This study used data from a primary study examining parent recommendations for adapting a digital health intervention for children’s pain, building on our previously developed app for adolescents (12–18 years) [15]. For both studies, we recruited participants in-person or through email (for clinicians) from the Division of Hematology/Oncology at the Hospital for Sick Children (SickKids) in Toronto, Ontario, Canada and the Hyundai Cancer Center at Children’s Hospital of Orange County (CHOC) in Orange, California, United States between December 2019 and September 2020. Available supports for parents at both centers include 24-7 phone access to an on-call clinician, which all families are made aware of at the onset of their child’s treatment. We enrolled a convenience sample of English-speaking parents of children aged 2–11 years currently receiving treatment for any cancer diagnosis. The upper limit of 11 years was selected as it aligned with our goal to adapt our existing adolescent-focused pain app for younger children (2–11 years; *PainCaRe* app) [10]. Two years of age was chosen as the lower boundary, as children younger than this often have extended periods hospitalized during treatment. Children were required to spend ≥ 25% of their treatment time outside of the hospital and to have experienced pain of any intensity in the previous week per parent report. We excluded parents of children receiving end-of-life care as these children may experience complex pain situations requiring specialized palliative care involvement and are frequently hospitalized during this period. Multidisciplinary pediatric oncology clinicians were eligible if they provided direct pain-related care to children with cancer. Clinicians were sampled using a snowball approach. Clinicians were recruited to offer a broad perspective on how parents manage pain at home, given their experience caring for many families and their training in recognizing pain in children with cancer. Clinicians may have cared for the children of parents enrolled in the study.

### 2.2. Data Collection

Following ethics approval at SickKids (#1000064500) and the University of California, Irvine (#190666), we explained the study and obtained written informed consent from participants. Trained research team members who did not personally know participants conducted one-on-one individual semi-structured interviews in-person, online using MS Teams, or over the telephone depending on participant preference. Field notes were taken and repeat interviews were not conducted. For the primary study, our participant interview guide was structured using two behaviour change–related theoretical models that address factors influencing app use and family caregiving capacity: (1) the Unified Theory of Acceptance and Use of Technology 2 (UTAUT2) framework [16] and (2) Van Houtven’s model of informal caregiver supports required to enable caregiving activities [17]. For the present analysis, we drew on participants’ responses to interview questions that aligned directly with the study objectives focused on children’s cancer pain and home-based pediatric cancer care, selected based on our clinical experience and prior research in these areas (selected questions are presented in Appendix B). Similar interview guides have successfully been used by our group to explore salient issues in home-based pediatric cancer care [5,18]. Interviews in the primary study were conducted until data saturation was reached. We used all available data for the present analysis. The present dataset has sufficient information power because the study aim was focused, participants were from specific and relevant groups with direct experience of childhood cancer pain, and the interviews yielded rich and detailed accounts, all of which increase the adequacy and depth of the data for addressing our research objective [19]. 

### 2.3. Data Analysis

Interviews were audio-recorded, transcribed, and uploaded to NVivo 12.0 for analysis. Descriptive statistics summarized participant characteristics. Using an inductive thematic analysis approach guided by the methods of Braun and Clarke [20], two authors who are experienced in qualitative methods (L.A.J. [pediatric oncology nurse researcher] and E.H. [research manager and expert in public health]) independently coded all data using a statement-by-statement approach with reference to the study objective. Coding decisions were reviewed regularly in meetings between L.A.J. and E.H., which continued throughout the data analysis period. Discrepancies in coding decisions were resolved through discussion between the two authors at these meetings. Generated codes were consolidated and grouped into themes and subthemes based on between-code relationships.

## 3. Results 

Twenty-one parents and 21 clinicians were approached, and all agreed to be interviewed. On average, parent interviews lasted 33 minutes (SD = 15, Range = 14–67). and clinician interviews also lasted 33 minutes (SD = 7, Range = 22–45). Participant characteristics are shown in Table 1. Most parents were between 30–39 years of age (62%; *n* = 13) and mothers (81%; *n* = 17). Clinicians were most often registered nurses (*n* = 9, 43%) and had an average of 10 years (SD = 9) of clinical experience. Table 2 presents themes and sub-themes with representative narrative quotes.

### 3.1. Theme 1: The Multifaceted Experience of Young Children’s Cancer Pain at Home

#### 3.1.1. “Whenever We Are Home, He Has Experienced Pain”: Pain Prevalence

Parents and clinicians reported that it is common for young children with cancer to experience pain at home. As evidence of the problematic nature of pain at home, clinicians reported pain-related emergency visits and calls to the healthcare team. One clinician reported these visits as, “[occurring] pretty frequently. Not all of them [children] get admitted, so they’ll have pain at home and then they manage on something oral” (Registered Nurse). However, it was noted that cancer pain presentations in young children differed from those in older children, making it sometimes difficult to detect pain. One clinician noted,

“So usually for the younger ones, the 2–7 [years olds] or so, many times their parents will say ‘They’re not playing, they’re laying around’. Or, if their stomach is hurting, they just don’t eat. So, a lot of times it’s like these subtle, you know just removed from their normal activity”.

(Advanced Practice Nurse)

#### 3.1.2. Physical Burden of Pain

Children’s cancer pain at home varied in severity from mild to severe, and its frequency ranged from daily to monthly episodes, suggesting a complex pattern of pain outside of the hospital. One parent remarked on the severity: “Yeah, he’ll cry. Before he’ll cry, he’ll speak out. Like ‘Mommy, I can’t eat, my tongue hurts, my throat hurts, I have pain here, my chest hurts’” (Mother of 6-year-old). Another parent described the duration of pain episodes: “In the best-case scenario, an hour. That’s the best-case scenario. With one chemo she was really having pain for a long time, like for days” (Mother, child age not reported). Common pain types included mucositis, abdominal discomfort, joint and muscle aches, as well as headaches. Such pain frequently co-occurred with fatigue. The causes of pain reported by parents and clinicians included neuropathy from chemotherapy, surgeries, and needle procedures. 

#### 3.1.3. Emotional and Psychological Impact

Pain contributed to increased anxiety in children. One clinician reported, “You know, the psychological has become the forefront for me as far as how kids perceive everything. There’s so much anxiety in our population…and then it goes from that anxiety, to fear, to provoked pain, to intensified pain” (Advanced Practice Nurse). Clinicians often observed a direct relationship between the levels of anxiety in a parent and their child, which then led to amplified or anticipatory pain in the child. Parents described negative pain-related changes in child mood and behaviour: “He would throw a tantrum if he was uncomfortable and didn’t really know how to say, ‘I’m uncomfortable’ or ‘This hurts.’ And he would cry or get frustrated” (Mother of 2-year-old child).

#### 3.1.4. “It Limits Her Ability to Participate”: Disruption to Daily Life and Childhood Activities

Pain was often described by both groups as interfering with typical childhood functioning. Specifically, negative impacts on eating, mobilizing, socializing with friends and family, and play-related behaviours were discussed by both clinicians and parents, with one clinician reporting, “So [the child] reports pain at home…it limits [their] ability to participate with family…and sometimes it even limits [the child’s] ability to eat” (Physical Therapist).

### 3.2. Theme 2: The Ripple Effects of a Young Child’s Cancer Pain on the Family

#### 3.2.1. Toll on Parents and Siblings

Young children’s cancer pain occurring at home was reported to be highly distressing for parents. Distress stemmed from uncertainty related to pain treatment methods, in particular which methods to use, what side effects they might have, the expected time before any analgesic effect, and when to escalate the pain issue to the level of the care team. One parent expressed: "Because I can’t imagine there’s a lot of parents out there that know what to do. A lot of us are experiencing these symptoms for the first time" (Mother of 2-year-old).

Parents described feelings of sadness and helplessness when observing their child in pain, especially in the initial stages following a cancer diagnosis. One parent stated,

“There’s just so much going on. [My child is] complaining a lot, you know, he used to say, ‘Make it stop.’ And you don’t know what to do. He complained of everything: headaches, legs feel like noodles, legs felt broken. And we don’t know what to do”.

(Stepmother of 10-year-old)

The impact of a young child’s cancer pain was also felt by siblings. One parent noted behavioral changes in their child when experiencing pain, including increased irritability toward their sibling: “Change in attitude…he’ll get aggressive, so aggression towards his brother.” (Mother, child age not reported).

#### 3.2.2. The Work of Pain-Related Care Coordination

Parents and clinicians described the central role parents assumed in coordinating their child’s pain care, with clinicians emphasizing this critical contribution to their child’s comfort. Some parents reported feeling ill-equipped for this work. One mother of a 2-year-old child noted,

“It’s stressful for us because our minds just start running and there’s no one. I can’t get a hold of anyone, I can’t call. I don’t know who to call if I’ve already called the one number I have. And I’ve been in that situation where I’m waiting for hours, hours, hours, days—and I don’t get that call.”

Clinicians acknowledged that parents faced challenges managing pain at home, but suggested that with appropriate education and guidance, parents could effectively fulfill this role.

#### 3.2.3. Impact of Pain Caregiving on Parental Wellbeing

Parents reported neglecting their own needs when providing pain care to their child:

“[…] I used to try to exercise but I struggle with feeling guilty. I shouldn’t but it’s just something I struggle with […] to like leave [my child] and go running, or something like that it just seemed my head was just somewhere else, it [exercising] wasn’t helping me.”.

(Mother of 5-year-old)

The negative impact of the child’s pain on parent autonomy and family functioning was also highlighted. Parents described being unable to engage in activities or roles due to child monitoring: "Honestly, I’m in the same room with her all the time. Because of her condition, she has muscle pain, and her balance is off, so I just always have to be keeping an eye on her, watching her” (Mother, child age not reported). Such accounts underscored the centrality of parent wellbeing, which both clinicians and parents recognized as critical to sustaining caregiving capacity and meeting the child’s needs.

### 3.3. Theme 3: Assessing and Treating Children’s Cancer Pain at Home

#### 3.3.1. “We Know the Symptoms”: Becoming Confident in Identifying Pain

Both groups of informants endorsed parents as experts at recognizing their child’s pain, “When [my child is] in pain, I can tell…Looking at [my child], I can tell. Even with [my child’s] facial expression, you can tell that something is not going on well” (Mother of 6-year old). When these changes were noticed in a child, parents described probing further: “When I notice a change in his expression, I’ll be like, ‘You don’t look okay. What’s going on?’ He’ll be like, ‘My mouth hurts when I’m eating, I can’t swallow” (Mother of 6-year-old). For younger and non-verbal children, behavioural cues including crying, yelling, and changes in sleeping habits and appetite were key pain indicators. 

At the same time, both parents and clinicians acknowledged that assessing pain in young children is inherently challenging due to limited self-reporting abilities. As one social worker noted, “I have lots of conversations with parents of young children who are concerned because [the child is] not vocal…Parents assume it’s pain but, with a 2-year-old, you don’t necessarily know…Is it pain, or is it nausea, or are they tired?” In practice, parents often used a process of elimination—providing food or naps first—to determine whether their child was experiencing pain. These strategies illustrated how parents developed growing expertise in recognizing subtle and complex signs of pain over time.

#### 3.3.2. Home-Based Pain Management Strategies

Parents reported using pharmacological, physical, and psychological pain treatment methods at home. Many parents reported relying first on non-pharmacological interventions—including massage, stretching, distraction, physical activity, hot and cold packs, breathing exercises, and baths—before pharmacological treatments. Some parents described a growing sense of confidence in their use of these methods, “But for me if [name of child] were to tell me [they are] in pain, I would try to give [my child] a bath, or rub [their] his legs, take [my child] on a walk before I just brought [them] in” (Mother of 5-year-old). Clinicians trusted parent capacity to appropriately treat their child’s pain: “I believe this—that our caregivers know their child best because they live it every day and come up with whatever routine or whatever [pain] treatment plan works” (Registered Nurse). In cases of unresolved pain, parents reported directly contacting their child’s healthcare team for support. This was also noted by clinicians who saw parents as vigilant and responsive to their child’s pain. 

#### 3.3.3. Communication and Collaboration with the Healthcare Team

Parents stressed the importance of having streamlined channels to communicate with their child’s healthcare team in case of pain and mechanisms to systematically track pain at home to facilitate these conversations with clinicians. While clinicians largely reported parents’ capacity to assess, triage, and relieve children’s pain favorably—evidenced by parents’ comfort in calling the hospital and low pain-related admission rates—they also reported instances where they were unaware of children’s pain at home until they queried the family at a subsequent hospital visit. One clinician provided an example: “We had one [child] that I just discharged today. They didn’t come in for pain, they came in for chemotherapy, but they had ongoing pain in [their] left and right legs, and it was after a lumbar puncture that they started to feel this” (Registered Nurse). Clinicians thus emphasized that hospital-based encounters were times for thorough pain assessment, with care plan adjustments made to better manage the pain at home if needed.

#### 3.3.4. Supports Needed to Optimize Home Pain Care

Many parents advocated for additional supports to manage pain: “When [we’re] at home, we don’t have the right resources to help them when they’re in pain” (Mother of 5-year-old). Several solutions were proposed by both participant groups. Clinicians discussed the need to empower, educate, and reinforce parents as the experts on their children. Providing additional education on pain physiology, age-appropriate validated pain assessment tools, and guidance on when to escalate pain issues to the healthcare team was suggested: “I think very clear guidelines from the medical team about like, what is expected pain, and what kind of is within acceptable or tolerable level, then, how to respond to that pain, and when to seek additional services” (Physical Therapist). A primary barrier noted by clinicians to providing this education was workload:

“Our unit is so busy. Clinic is busy, [inpatient] is busy, [outpatient] is busy. We’re always so busy and I feel like that’s the biggest gap. All the nurses and the doctors and pharmacists are great at doing education. But it’s just so busy that sometimes we don’t have the time to do as much education as we want. That’s the biggest thing”.

(Physical Therapist)

Support groups were identified by both groups as valuable resources for parents, allowing them to connect with families with similar experiences: “Sometimes it really is helpful to know what’s worked for other people for similar pain” (Advanced Practice Nurse). Additionally, parents suggested that home-based relaxation resources could help manage their own stress: “I did really struggle with stress, so it was using the Insight timer and getting these breath sessions that I can use that led me to explore Instagram and find more of my favorite breath coaches to help me with deep breathing and stress management” (Mother of 5-year-old).

Developing coping strategies for parents was considered by both groups as needed to support children’s pain management at home, but also to enable adjustment to the cancer diagnosis in general: “Like, pain is one aspect of this…but if there’s a way to understand what [coping strategies the family have], and what’s working and what hasn’t, that may help [the family]” (Social Worker).

## 4. Discussion

Our study highlights the burden that managing young children’s cancer pain at home can place on families and the pressing need for enhanced supports for parents in this role. Both parents and clinicians acknowledged the prevalence of young children’s cancer pain outside of the hospital setting and identified key barriers to effective pain management. These findings underscore the need for systematic interventions to improve home-based pain care for young children with cancer.

Parents play a critical role in recognizing and addressing their child’s pain, yet some expressed uncertainties about appropriate methods to assess and treat it. Clinicians generally described parents’ abilities to assess and respond to pain favorably, but also noted that pain sometimes went unreported to them until elicited by them at a hospital visit. Together, these findings highlight two complementary challenges: parents’ need for greater guidance in pain management and the need for structured communication strategies to support timely reporting of pain. Previous studies have shown that standardized pain education can improve parental confidence and pain management outcomes [21]. Our findings reinforce the importance of equipping parents with evidence-based pain care strategies [10,22], particularly for young children who may have difficulty verbally expressing their pain and in light of known communication barriers in pediatric oncology [23,24]. When child self-report is not reliable, behavioral assessment measures provide an essential means of evaluating pain. Evidence-based tools, such as the Face, Legs, Activity, Cry, Consolability (FLACC) and revised-FLACC scales, have been shown to accurately assess pain in young children [25,26] and their use at home should be recommended to trained parents to guide timely pain management.

Our results also indicate that managing children with cancer pain at home can be emotionally distressing for parents, who often felt unprepared for the task. Uncertainty about when to escalate care, fear of undertreating or overtreating pain, and difficulty accessing timely guidance from clinicians compounded this stress and resulted in parents not attending to their own physical and psychological needs. These findings align with prior research demonstrating that parents of children with cancer experience high levels of caregiving burden and distress and that this distress may impact on child wellness [27,28,29]. Standards for clinical practice recommend preventing and treating children’s cancer pain as well as providing routine psychosocial support to families, both of which may help to alleviate some of these challenges [30,31].

Clinicians in our study noted that workload constraints limit the pain education they can provide to parents, reflecting broader challenges in pediatric oncology where time and resources for family-centered education are often scarce [32]. At the same time, clinicians themselves report receiving minimal training in biopsychosocial pain assessment management and having limited access to pain teams and high clinical demands [32,33,34,35]. Adjunct approaches—such as digital training modules for families, mobile apps, and standardized discharge protocols that emphasize pain care practices—may help address these gaps. Support groups also offer a valuable avenue for peer-to-peer advice, providing emotional support and practical, experience-based strategies.

However, our findings also highlight a broader issue: the “critical role” parents play in managing young children’s cancer pain at home often constitutes invisible work. Stemming from a sense of duty and concern, parents frequently assume substantial care responsibility, potentially leading to burden and distress [36]. From a family-centered care perspective, part of the clinicians’ responsibility is to protect the roles of children and parents, ensuring that families are supported rather than relied upon to fill gaps in care. While educating parents on recognizing pain and administering interventions remains essential, clinicians and institutions must also provide direct support for pediatric pain management. System-level strategies may include home-based nursing support [5,37], structured symptom assessment schedules [38] and sufficient staffing [39,40] to enable critical tasks.

Study strengths include that we utilized a dual-site design and was conducted across Canada and the United States. Despite differences in healthcare systems, parents and clinicians in both settings described similar challenges in managing pain at home, suggesting these issues reflect broader gaps in pediatric cancer care rather than center-specific issues. Future research could explore how structural differences, such as access to home care or coverage for supportive services, shape family experiences and inform scalable interventions. Additionally, by including both parents and multidisciplinary pediatric oncology clinicians, the study captures multiple viewpoints on young children’s pain at home and enabled the triangulation of our themes. Despite strengths, several limitations should be acknowledged. First, the study was conducted in specialized pediatric oncology centers and with parents who speak English (the dominant language of both care centers), which may limit the applicability of findings to other settings and families, particularly those with fewer resources. Second, we included only parents of children aged 2–11 years, reflecting the focus of our primary study to adapt a pain management app for younger children. As such, the experiences of parents of older children were not captured, despite their likely ongoing role in pain management. Third, data collection took place during the COVID-19 pandemic, which may have influenced participants’ experiences with healthcare access, communication, and emotional stress. Fourth, we did not conduct member checking of our themes and thus cannot comment on participants’ perceptions of their accuracy. 

Managing cancer pain in young children at home can be a complex and emotionally demanding task for parents. Our study underscores the need for improved pain education for both parents and clinicians, direct pain support by clinical teams, streamlined communication pathways between families and their healthcare teams, and expanded access to peer support networks. By addressing these gaps, clinicians and health systems can better support families in managing their children’s pain, ultimately improving outcomes for children and their families. Policy and practice implications from this work include directives to integrate structured pain education into routine pediatric oncology care and develop standardized communication pathways between families and care teams. Further, as a direct extension of this work, our own team is developing the *PainCaRe* app [10], a digital health intervention that provides parents with real-time support for managing their children’s pain at home and facilitates communication with oncology teams. A pilot randomized controlled trial (ClinicalTrials.gov Identifier: NCT06651190) will be launched across the two centers in this study and will identify barriers and facilitators to implementing communication and education strategies. Together, these findings can inform how such strategies can be adapted and scaled across oncology centers to strengthen family-centered pain care.

## Figures and Tables

**Table 1:** Participant characteristics.

Characteristic	N (%)	M (SD)
**Parents**		
Caregiver type		
Mother	17 (81)	
Father	3 (14)	
Stepmother	1 (5)	
Marital status		
Married	19 (90)	
Divorced	1 (5)	
Single	1 (5)	
Sex		
Female	18 (86)	
Male	3 (14)	
Age range (years)		
20–29	3 (14)	
30–39	13 (62)	
40–49	5 (24)	
Parent-identified race or ethnicity		
White	8 (38)	
Hispanic/Latinx	5 (24)	
Chinese	2 (10)	
South Asian	2 (10)	
Arab	1 (5)	
Armenian	1 (5)	
Black	1 (5)	
Korean	1 (5)	
Highest level of education		
College/University	9 (43)	
Professional School/Graduate Degree	7 (33)	
High School	4 (19)	
Teaching Credential	1 (5)	
Primary language		
English	16 (76)	
Arabic	1 (5)	
Cantonese	1 (5)	
Korean	1 (5)	
Tamil	1 (5)	
Urdu	1 (5)	
Child age (years)		5.3 (2.7)
Child cancer diagnosis		
ALL	9 (43)	
AML	4 (19)	
LCH	2 (10)	
Neuroblastoma	2 (10)	
APML	1 (5)	
Lymphoma	1 (5)	
MYH9-related disorder	1 (5)	
Osteosarcoma	1 (5)	
Time since child diagnosis (years)		0.9 (1.4)
**Clinicians**		
Profession		
Advanced Practice Nurse	5 (24)	
Oncologist	1 (5)	
Physical Therapist	3 (14)	
Psychologist	1 (5)	
Registered Nurse	9 (43)	
Social Worker	2 (10)	
Sex		
Female	21 (100)	
Male	0 (0)	
Age range (years)		
20–29	7 (33)	
30–39	8 (38)	
40–49	5 (24)	
50–59	1 (5)	
Highest level of education		
Bachelor’s Degree	7 (33)	
Master’s Degree	10 (48)	
Doctoral Degree	3 (14)	
Medical Degree	1 (5)	
Clinical work experience (years)		10 (9)
Pediatric oncology clinical work experience (years)		8 (8)

ALL, acute lymphoblastic leukemia; AML, acute myeloid leukemia; LCH, Langerhans cell histiocytosis; APML, acute promyelocytic leukemia.

**Table 2:** Thematic analysis framework with quotes by participant group.

Theme		Sub-Theme	Parent Representative Quote	Clinician Representative Quote
1	**The Multifaceted Experience of Young Children’s Cancer Pain at Home**	***“Whenever we are home, he has experienced pain”: Pain Prevalence***	“So, whenever we are home, yeah, he has experienced pain. Like, it has been fifteen months into this experience, so we know”. (Mother, child age not reported)	“Oh gosh, I feel that it occurs pretty frequently. Not all of them get admitted. Sometimes they’ll have pain at home and then they manage on something oral. I feel like the times that I [a child in pain is] maybe once a week where they actually get admitted. But the amount of kids that go through [the emergency department] is probably more than that.” (Registered Nurse)
		***Physical Burden of Pain***	“She has her disease in her bones so…she has [pain] in her arms and her hip bone, liver, and spleen. So sometimes she says that her tummy hurts, but I’m not sure if it’s directly related”. (Mother, child age not reported)	“[Parents will come back to the hospital and say, ‘He has pain in his leg’ but then that was basically all we got. And in the younger population their memory and recall isn’t great so then the caregiver is trying to remember.” (Registered Nurse)
		***Emotional and Psychological Impact***	“It promoted more language out of him. And so at that point, he would complain if he wasn’t feeling right or sometimes”. (Mother of 2-year-old child)	“I think sometimes the parents or the caregivers need to observationally see what the patient is doing and how they’re reacting to mom’s touch on that area, or if it’s not bothering them, but they’re saying it’s bothering them. Because I’ve seen that a lot too, where there’s that fear/anxiety kind of component.” (Physical Therapist)
		***“It limits her ability to participate”: Disruption to Daily Life and Childhood Activities***	“I’ve noticed she has pain more when she’s more active like more playful and walking more. That’s when I’ll notice that she has pain”. (Mother of 7-year-old child)	“So usually for the younger ones, I would say 2–7 [years old] or so, their parents will tell that they’re not playing. They say, ‘They’re not playing, they’re lying around.’ So, a lot of times it’s like these subtle, you know just removed from their normal activity.” (Advanced Practice Nurse)
2	**The Ripple Effects of a Young Child’s Cancer Pain on the Family**	***Toll on Parents and Siblings***	“Right, because I’m already on edge. We’ve been through so much. And [my child’s pain is] going to scare me from now on and I think it’s just because of our situation”. (Mother of 4-year-old child)	“It’s very distressing for parents to have a child with an illness and then also experiencing pain on top of that.” (Physical Therapist)
		***The Work of Pain-Related Care Coordination***	“Then of course we have to call here [the hospital] first. But I don’t know if we’re still allowed to call after we’re discharged.” (Father of 5-year-old child)	“I think that [parents] have a really hard time, especially when they are used to being in the hospital and they got a lot of help here.” (Registered Nurse)
		***Impact of Pain Caregiving on Parental Wellbeing***	“Right now, I am thirty and I’m turning thirty-one this year. And lots of my friends are getting married now. Some of them are having kids at the moment. So, I feel like I’m being left out” (Mother of 7-year-old child).	“Or, maybe they’re just not coping. Like, pain is one aspect of this but the family is not coping through any of this so if there’s a way to understand what they’ve tried, and what’s working and what hasn’t, that may help.” (Social Worker)
3	**Assessing and Treating Children’s Cancer Pain at Home**	***“We know the symptoms”: Becoming Confident in Identifying Pain***	“Even If he doesn’t tell you, just looking at him, you can figure it out. What I do is say, “Are you okay?”. He’ll be like,‘No’ and I know”. (Mother of 2-year-old child) “I mean it would be helpful to have a better communication with her but it’s hard because of her age to know how much pain she really is experiencing”. (Mother of 5-year-old child)	“I feel like the parents are really good at knowing when their kids in a lot of pain or there just a little. They just know the signs to look for their kids.” (Registered Nurse) We don’t necessarily teach parents about that. So, I think it becomes more behavioural for younger kids. Which is definitely difficult to do because sometimes kids are crying and inconsolable, and that can be indicating pain but it also can be indicating [that] they’re just cranky, or they have a fever, or they want a cookie, I don’t know. It’s not necessarily so cut and dry when they’re younger.” (Registered Nurse) “I’ve seen a few parents ask their kids like out of then, what is your pain right now and the kids will answer. I guess it depends on the age and like the cognitive abilities of the kids too.” (Registered Nurse)
		***Home-Based Pain Management Strategies***	“[There is an] app that he uses, the hospital introduced us to [it], and that was a really good distraction—especially when he was managing pain as a baby”. (Mother of 7-year-old child)	“[Parents will let] them watch their movies, listen to music or tablets. Doing things like talking, doing things their kids like, any distractions, and giving them comfort items…Using family, [parents will] bring families members that [the child finds] comfort in.” (Social Worker)
		***Communication and Collaboration with the Healthcare Team***	“I think it’s nice to have the ability to like to be able to like even just be able to send an email of like this is what’s going on right now [to the team].” (Father, child age not reported)	“Impowering [parents] to communicate with us, whether it’s to really observe their [child’s] symptoms or to identify a plan to co-produce with us.” (Social Worker)
		***Supports Needed to Optimize Home Pain Care***	“If the hospital is aware [of my child’s pain], if the doctors, the nurses, or any healthcare professional are aware of that, that would be helpful.” (Mother of 6-year-old child)	“Call us and help us [clinicians] make a plan so that we can come up with step A, B, C as opposed to waiting until the pain is so severe that they [the child and parent] have to go to the [Emergency Department]. We want to ultimately just empower them to be able to manage the pain at home so that we don’t get a call when it’s a 9/10 and they end up in the [Emergency Department].” (Social Worker)

## Data Availability

Data from this study are available (in accordance with institutional policies) from the corresponding author upon request.

## Abbreviations

CHOC	Children’s Hospital of Orange County
SickKids	The Hospital for Sick Children

## Appendix A

Manuscript associated consolidated criteria for reporting qualitative studies (COREQ) checklist.

Developed from:

Tong A, Sainsbury P, Craig J. Consolidated criteria for reporting qualitative research (COREQ): a 32-item checklist for interviews and focus groups. International Journal for Quality in Health Care. 2007. Volume 19, Number 6: pp. 349–357 [12].

**Item No**	**Guide Questions/Description**	**Reported on Page #**
**Domain 1: Research team and reflexivity**		
***Personal Characteristics***		
1. Interviewer/facilitator	Which author/s conducted the interview or focus group?	p. 3 (“Trained research team members who did not personally know participants conducted individual semi-structured interviews”)
2. Credentials	What were the researcher’s credentials? (e.g., PhD, MD)	p. 1
3. Occupation	What was their occupation at the time of the study?	p. 3 (“(L.A.J. pediatric oncology nurse researcher”)
4. Gender	Was the researcher male or female?	Female
5. Experience and training	What experience or training did the researcher have?	p. 3 (“two authors who are experienced in qualitative methods (L.A.J. [pediatric oncology nurse researcher] and E.H. [research manager and expert in public health])”)
***Relationship with participants***		
6. Relationship established	Was a relationship established prior to study commencement?	p. 3 (“Trained research team members who did not personally know participants conducted individual semi-structured interviews”)
7. Participant knowledge of the interviewer	What did the participants know about the researcher?	p. 3 (“Trained research team members who did not personally know participants conducted…interviews”)
8. Interviewer characteristics	What characteristics were reported about the interviewer/facilitator?	p. 3. (“University of California, Irvine (#190666), we explained the study and obtained written informed consent from participants”)
**Domain 2: Study design**		
***Theoretical framework***		
9. Methodological orientation and theory	What methodological orientation was stated?	p. 2 (“interpretive descriptive qualitative design”)
***Participant selection***		
10. Sampling	How were participants selected?	p. 2 (“convenience sample of English-speaking parents…” and “clinicians were sampled using a snowball approach”)
11. Method of approach	How were participants approached?	p. 2 (“recruited participants in-person or through email…between December 2019 and September 2020.”)
12. Sample size	How many participants were in the study?	p. 3 (“21 parents and 21 clinicians participated”)
13. Non-participation	How many refused or dropped out?	p. 3 (“Twenty-one parents and 21 clinicians were approached, and all agreed to participate”)
14. Setting of data collection	Where was the data collected?	p. 3 (“interviews in-person, online using MS Teams, or over the telephone depending on participant preference”)
15. Presence of non-participants	Was anyone else present?	p. 3. (“conducted one-on-one individual semi-structured interviews”)
16. Description of sample	Important sample characteristics?	p. 3–5 (Table 1: demographics of parents and clinicians)
***Data collection***		
17. Interview guide	Were guides provided? Pilot tested?	p. 3 (“semi-structured interview guide… structured using two theoretical models… based on those…used by our group to explore salient issues in home-based pediatric cancer care, Appendix A”)
18. Repeat interviews	Were repeat interviews carried out?	p. 3 (“repeat interviews were not conducted”)
19. Audio/visual recording	Did they use recording?	p. 3 (“Interviews were audio-recorded”)
20. Field notes	Were field notes made?	p. 3 (“Field notes were taken)
21. Duration	Duration of interviews/focus group?	p. 3 (“interviews lasted 33 minutes)
22. Data saturation	Was data saturation discussed?	p. 3 (“Interviews… were conducted until data saturation was reached”)
23. Transcripts returned	Were transcripts returned to participants?	p. 11 (“we did not conduct member checking…”)
**Domain 3: Analysis and findings**		
***Data Analysis***		
24. Number of data coders	How many coded the data?	p. 3 (“two authors… independently coded all data”)
25. Description of coding tree	Was a coding tree described?	N/A (“coded all data using a statement-by-statement approach”)
26. Derivation of themes	Were themes pre-identified or from data?	p. 3 (“inductive thematic analysis… generated codes were consolidated into themes”)
27. Software	What software used?	p. 5 (“uploaded to NVivo 12.0”)
28. Participant checking	Did participants give feedback on findings?	p. 11 (“we did not conduct member checking of our themes”)
***Reporting***		
29. Quotations presented	Were participant quotes presented?	p. 7–13 (Table 2 and Results section with quotes)
30. Data and findings consistent	Was there consistency between data and findings?	Yes, demonstrated across Results section (pp. 7–13)
31. Clarity of major themes	Were major themes clearly presented?	p. 7–10 (Themes 1–3 explicitly reported)
32. Clarity of minor themes	Were minor themes/diverse cases described?	p. 7–10 (subthemes and diverse perspectives included)

## Appendix B

Semi-Structured Interview Guide.
**Parent interview guide**
Possible probes: - *Can you tell me more about that?* - *Can you tell me why that is true (not true) for you?* - *What might make you think differently about that?* - *Can you give me an example?* - *How was this different from what you expected?*
Child pain experiences at home **1.** Can you tell me about some times that your child experienced pain at home? **2.** How did you know they were experiencing pain? **3.** How often does your child have pain at home? **4.** How long does the pain last when it happens? **5.** How often is your child’s pain related to cancer? **6.** How often is your child’s pain related to cancer treatments like chemotherapy, radiation or surgery? **7.** How often is your child’s pain related to cancer procedures like needle pokes? **8.** How confident are you that you can tell when you child has pain? **9.** What would help you to cope with your child’s pain better when they are not at the hospital?
Current management strategies **10.** When your child has pain at home, what things do you do to make them feel better? **11.** Do you try medications? Why or why not? **12.** Do you try things like distraction or relaxation? Why or why not? **13.** Do you try things like stretching or walking? Why or why not? **14.** How well do the things you do at home work? **15.** Is there anything that would help them work better?
Interview conclusion Is there anything else you wanted to tell me about your child’s cancer pain that we have not discussed today? Thank you very much for participating in this study. The information you have provided is invaluable to use as well refine and develop an app for parents providing pain management to their young children with cancer.
**Clinician interview guide**
Possible probes: - *Can you tell me more about that?* - *Can you tell me why that is true (not true) for you?* - *What might make you think differently about that?* - *Can you give me an example?* - *How was this different from what you expected?*
Child pain experiences at home **1.** Can you tell me about a time that caregivers or children under your care experienced pain at home? **2.** How did you find out they were experiencing pain? **3.** How often did it occur? **4.** How long did it last? **5.** What was it due to? **6.** Do you find that caregivers are able to assess and manage pain in their children outside of the hospital? **7.** How should caregivers be supported to assess and manage pain in their children outside of the hospital?
Current management strategies **8.** What pain management strategies do caregivers report to you they are using with their children when at home? **9.** How effective are the strategies caregiver do at home? **10.** Is there anything that would help these strategies work better?
Interview conclusion Is there anything else you wanted to tell me about your perceptions of child’s cancer pain that we have not discussed today? Thank you very much for participating in this study. The information you have provided is invaluable to use as well refine and develop an app for parents providing pain management to their young children with cancer.
